# TIME-TO-EVENT ANALYSIS IN EVALUATING THE ASSOCIATION OF FALL RISK APPRAISALS AND PHYSICAL ACTIVITY IN OLDER ADULTS

**DOI:** 10.1093/geroni/igae098.3292

**Published:** 2024-12-31

**Authors:** Tho Nguyen, Ladda Thiamwong, Chang Liu, Jethro Raphael Suarez, Joon-Hyuk Park, Rui Xie

**Affiliations:** University of Central Florida, Orlando, Florida, United States; University of Central Florida, Orlando, Florida, United States; University of Central Florida, Orlando, Florida, United States; University of Central Florida; University of Central Florida, Orlando, Florida, United States; University of Central Florida, Orlando, Florida, United States

## Abstract

Older adults are encouraged to participate in moderate-to-vigorous physical activity (MVPA) for 75 to 300 minutes per week for health benefits. However, the association of fall risk appraisal (FRA) with meeting the recommended activity levels remains uncertain. We assessed FRA in 134 participants aged 61 to 89 (mean age=74.26±6.85 years, 88.8% female, and 70.15% no history of falls) using the Short Fall Efficacy Scale-International and a static balance assessment, and categorized them into four FRA groups: rational (10.5%, low fear of falling (FOF) and normal balance), irrational (42.9%, high FOF but normal balance), incongruent (25.6%, low FOF and poor balance), and congruent (21.1%, high FOF but poor balance). Using time-to-event analysis based on daily accelerometer activity count data, we examined the impact of FRA on the duration of MVPA, light physical activity (LPA), and sedentary behavior (SB) across seven consecutive days. Kaplan-Meier curves demonstrate that older adults in the rational FRA group had the highest probability of sustaining MVPA throughout the day, whereas those in the congruent group had the lowest probability (log-rank p < 0.001). Conversely, participants in the congruent group exhibited the highest likelihood of extending SB duration, while those in the rational group had the lowest (log-rank p < 0.001). No significant differences were observed among FRA groups regarding LPA duration. The findings underscore the significance of mitigating a sedentary lifestyle and emphasize the importance of implementing targeted interventions to enhance the duration of MVPA as a pivotal determinant in diminishing the fall risks among older adults.

